# Supplementary material for: Gene drive escape from resistance depends on mechanism and ecology
Source: Evol Appl. 2022 Mar 22;15(5):721–34. doi: 10.1111/eva.13358 (PMC9108321; doi:10.1111/eva.13358)
Supplement: Supplementary file 1 — Supplement S1 [file EVA-15-721-s001.pdf]

Appendix: Impact of Inbreeding on a Homing Gene Drive with Deleterious Fitness Effects

In this appendix, we analyze how inbreeding affects a 2-sex homing gene drive with recessive deleterious effects on fitness.

Let P_{AA}, P_{Aa}, P_{aa} be the frequencies of the drive locus genotypes at the start of a generation. The frequencies of the drive A and wildtype a alleles are, respectively $p = P_{AA} + P_{Aa}/2$ and $q = 1 - p$. The inbreeding coefficient is f.

Selection

The fitness of AA is $1 - s$. After selection, genotype frequencies change to $P^*_{AA} = P_{AA}(1 - s)/\bar{w}$, $P^*_{Aa} = P_{Aa}/\bar{w}$, and $P^*_{aa} = P_{aa}/\bar{w}$ where

$$\begin{aligned}\bar{w} &= P_{AA} \cdot (1 - s) + P_{Aa} \cdot 1 + P_{aa} \cdot 1 \\ &= 1 - sP_{AA} \\ &= 1 - sp(p + fq).\end{aligned}$$

The drive allele response to selection is thus

$$\begin{aligned}\Delta^{\text{sel}} p &= p^* - p \\ &= P^*_{AA} + \tfrac{1}{2} P^*_{Aa} - p \\ &= \frac{-sP_{AA} \cdot q}{\bar{w}} \\ &= \frac{-spq(p + fq)}{1 - sp(p + fq)}\end{aligned}$$

Segregation distortion

The advantage gained by the drive via segregation distortion with distortion parameter δ is

$$\begin{aligned}\Delta^{\text{sd}} p &= \delta P^*_{Aa} \\ &= \frac{\delta P_{Aa}}{\bar{w}} \\ &= \frac{\delta 2pq(1 - f)}{1 - sp(p + fq)}.\end{aligned}$$

Equilibria

At equilibrium, the change from selection must offset that from segregration distortion, that is, $|\Delta^{\text{sel}} p| = |\Delta^{\text{sd}} p|$. Solving this condition for p gives the

equilibrium drive allele frequency as a function of δ, the segregation distortion parameter, s, the selection coefficient against drive homozygotes, and f the inbreeding level:

$$\hat{p} = \frac{2\delta}{s} - \frac{f}{1-f}.$$

In the main text, when the Q allele is fixed, the inbreeding coefficient is equivalent to $f = m$ and the corresponding drive equilibrium is

$$\hat{p} = \frac{2\delta}{s} - \frac{m}{1-m}.$$

With complete distortion ($\delta = 1/2$), this expression simplifies further to

$$\hat{p} = \frac{1}{s} - \frac{m}{1-m}.$$

These equilibria are biologically feasible if and only if $0 \leq \hat{p} \leq 1$.
